# Associations of Prior Chronic Use of Non-Steroidal Anti-Inflammatory Drugs (NSAIDs) and Glucocorticoids With Cachexia Incidence and Survival

**DOI:** 10.3389/fonc.2022.922418

**Published:** 2022-06-07

**Authors:** Santiago Olaechea, Anne Gilmore, Christian Alvarez, Bhavani S. Gannavarapu, Rodney Infante, Puneeth Iyengar

**Affiliations:** ^1^ Center for Human Nutrition, University of Texas (UT) Southwestern Medical Center, Dallas, TX, United States; ^2^ Department of Clinical Nutrition, University of Texas (UT) Southwestern Medical Center, Dallas, TX, United States; ^3^ Department of Radiation Oncology, University of Texas (UT) Southwestern Medical Center, Dallas, TX, United States

**Keywords:** cachexia, NSAIDs (non-steroidal anti-inflammatory drugs), glucocorticoids, weight loss, palliative care, drug repurposing and discovery

## Abstract

**Background:**

Cachexia is an inflammatory and metabolic syndrome of unintentional weight loss through depletion of muscle and adipose tissue. There is limited knowledge of how chronic use of non-steroidal anti-inflammatory drugs (NSAIDs) and glucocorticoids affect cachexia development. The purpose of this study was to investigate associations between prior long-term use of NSAIDs or glucocorticoids with cachexia incidence and post-diagnosis weight loss progression in a retrospective cancer patient cohort.

**Methods:**

Of 3,802 lung or gastrointestinal cancer patient records, 3,180 comprised our final cohort. Patient demographic information, tumor qualities, medication histories, and comorbidities were assessed. Cachexia was defined as having developed prior to oncologic treatment. Statistical evaluations included categorical, multivariate logistic regression, and log-rank survival analyses. Development of substantial post-diagnosis weight loss was calculated and interpreted for patients without cachexia at diagnosis.

**Results:**

Chronic prior use of any NSAID or glucocorticoid medication was associated with approximate absolute and relative reductions in cachexia incidence at diagnosis of 10 and 25 percent (*P<0.0001*). In multivariate analyses, NSAID medications demonstrated a 23 percent reduction in cachexia incidence likelihood (OR=0.770; 95% CI=0.594, 0.998; *P=0.0481*). Patients without cachexia at diagnosis were significantly more likely to develop substantial post-diagnosis weight loss from pre-diagnosis use groups of glucocorticoids (OR= 1.452; 95% CI=1.065, 1.979; *P=0.0183*) or NSAIDs (OR=1.411; 95% CI=1.082, 1.840; *P=0.011*).

**Conclusions:**

Our findings suggest a protective effect of prior anti-inflammatory medications, primarily NSAIDs, against manifestations of the cachexia phenotype at cancer diagnosis. These observations support further exploration of potential therapeutic benefits from anti-inflammatory medications early in cancer management.

## Introduction

Cancer-associated wasting, known as cancer cachexia, poses a significant challenge in the management of patients across many primary malignancies. While weight loss is the hallmark of this syndrome, cachexia is further characterized by maladaptive inflammatory signaling through modulation of pathways including JAK/STAT (often through IL-6 cytokines) and both NF-kB and MAP kinase (through TNF-α induction) (1–4). These tumor-directed, cytokine-dependent mechanisms help set apart cachectic weight loss from weight loss attributable to alternative processes such as treatment induced anorexia and dysphagia. Developments within the field have further implicated cancer cachexia with reduced physical function, loss of appetite, sarcopenia, fatigue, lower self-reported quality of life scores, increased treatment-related toxicity, and worse survival outcomes (5, 6). Currently, physicians have few pharmacological interventions available for cachexia-specific management. Available therapies primarily address nutritional consequences without targeting the maladaptive systemic changes underpinning cachectic pathophysiology (7).

Multiple pre-clinical and clinical trials have suggested potential benefits in application of non-steroidal anti-inflammatory drugs (NSAIDs) and glucocorticoid medications in the treatment of cachexia-associated weight loss (5, 8–11). The mechanistic premise for NSAID use is their COX inhibition, which impairs the production of prostaglandins known to contribute to inflammation and tumor progression. In addition to appetite stimulation, corticosteroids alter gene expression with significant downstream anti-inflammatory effects, such as interference with NF-κB activation (12). Cohort characteristics, treatment groups, and outcomes of notable studies are summarized in **Supplementary Table 1**.

The aforementioned trials have been insufficient to validate the inclusion of NSAIDs or glucocorticoids in cachexia treatment guidelines (7, 13, 14). Of note, these trials have almost exclusively included patients with advanced cancer, where the degree of cachexia might be exceedingly difficult to overcome with treatment. In these cohorts, patients have already undergone surgical, chemotherapeutic, hormonal, immunologic, or radiation treatments, most of which are known to introduce various alternative mechanisms for weight loss outside the metabolic paraneoplastic effects of cachexia. In contrast, our current effort in this study focuses on patient use of daily medications with anti-inflammatory properties prior to their cancer diagnosis. The novel emphasis on cachexia and use of these medications use prior to a cancer diagnosis and tumor-directed treatment allows for a better understanding of the effects of these medications on the development of cachectic weight loss specifically attributable to the metabolic and inflammatory alterations induced by the tumor itself on host tissues. Fundamentally, the purpose of this study was to determine the association between prior long-term use of NSAIDs or glucocorticoids with cachexia incidence at the time of cancer diagnosis and their effects on cachexia and non-cachexia associated survival.

## Materials and Methods

### Patient Cohort

**Figure 1:**
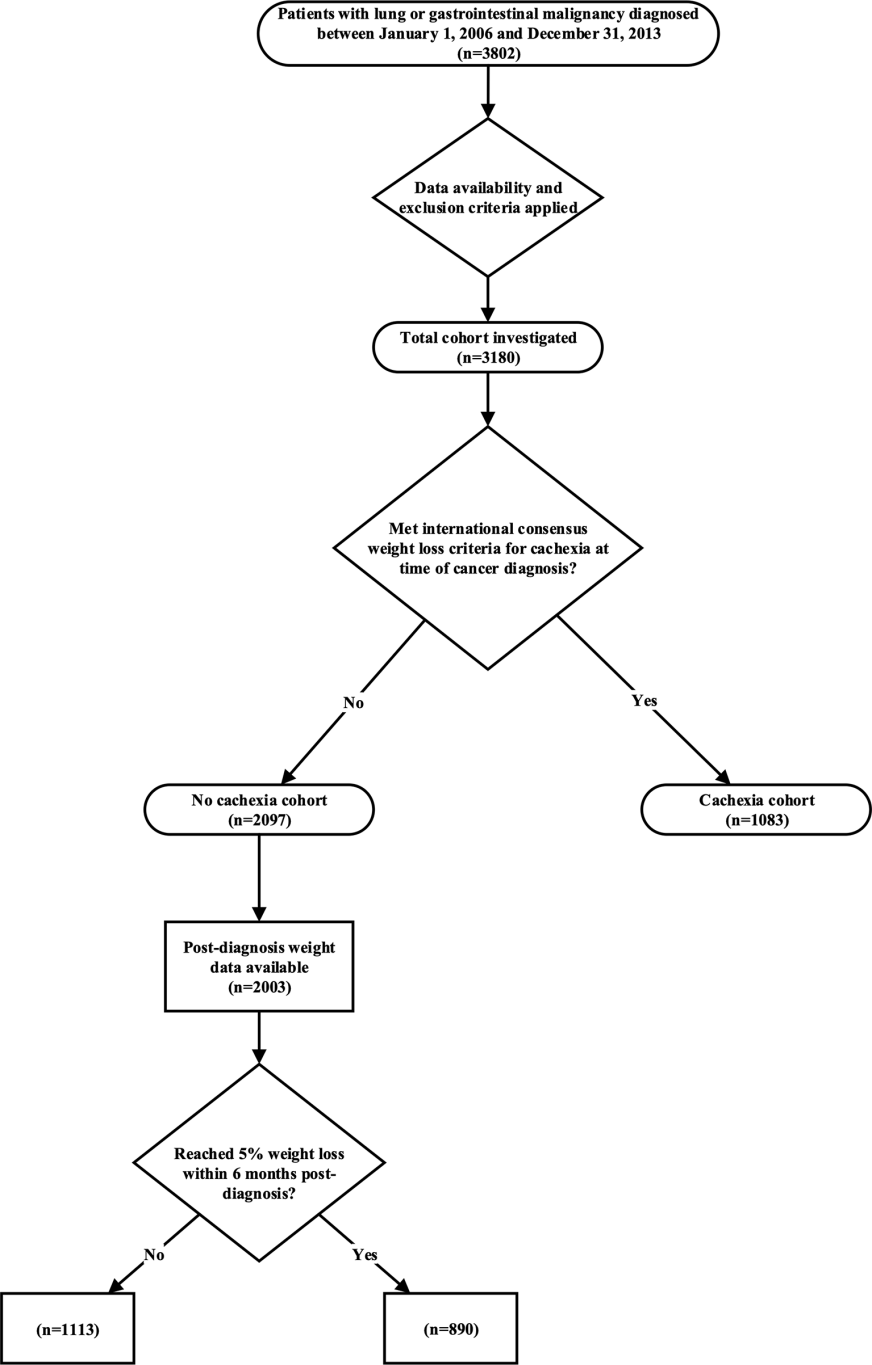
Cohort selection and stratification diagram.

**Figure 1** demonstrates our cohort selection and stratification process. Using the clinical research data warehouse at UT Southwestern Medical Center and supplemental chart review for validation and data collection, we identified 3,802 patients with lung or gastrointestinal cancer diagnosed between 1/1/2006 and 12/31/2013 for evaluation. Patients were excluded if records were incomplete or if the classification of tumor histology was carcinoma in situ, sarcoma, or melanoma. After applying exclusion criteria, 3,180 patients were eligible for the study database. Demographic and tumor characteristics from the time of cancer diagnosis were recorded, and a calculation of each patient’s Charlson Comorbidity Index (15) was carried out to demonstrate their comorbidity risk. The UT Southwestern Medical Center Institutional Review Board approved this study.

### Determination of Cachectic Weight Loss at Diagnosis and Significant Post-Diagnosis Weight Loss

Cachexia was retrospectively evaluated for the total cohort through review of patient medical records in which weight measurements were routinely documented at each office visit. The assessment of cachexia for each patient was supplemented by vital signs, physician notes, and dietitian notes specifically at the time of cancer diagnosis but before any treatment had been administered. The international consensus definition of cancer cachexia served as the basis for our classification of cachexia at diagnosis (16). In patients with a BMI ≥ 20 kg/m^2^, unintentional weight loss greater than 5% of total weight within 6 months prior to cancer diagnosis led to a classification of demonstrating cachectic pre-treatment weight loss. In patients with a BMI < 20 kg/m^2^, unintentional weight loss greater than 2% within 6 months prior to cancer diagnosis led to the same classification. Patients with stable weight, purposeful weight loss, or weight gain were not categorized as having cachectic weight loss.

To define significant weight loss in patients who did not present with cachexia at diagnosis, we applied criterion of reaching 5% weight loss within any 6-month period that ended within 6 months post-diagnosis.

### Defining Prior Anti-Inflammatory Medication Use

Medication prescription and administration logs were obtained through extraction of health system medical records. Patients were placed into prior medication use groups if they were prescribed or administered the selected medications for chronic use prior to cancer diagnosis. We defined chronic use medications as daily medications started at least 3 months before the cancer diagnosis date. Stringent criteria were applied in the selection of medication dosage and formulation type, and any acute or subacute intervention was excluded. Median time from initial medication use per patient to their cancer diagnosis was calculated.

### Statistical Analysis

Chi square testing determined categorical associations between prior medication use groups and cachexia at diagnosis, as both were recorded as binary variables for each patient. The primary control group consisted of patients without chronic use of anti-inflammatory medications (specifically NSAIDs and glucocorticoids in this study) prior to their cancer diagnosis. An additional control group, patients positive for chronic metformin use and negative for chronic anti-inflammatory medication use, was included in categorical testing. Metformin was selected due to its classification as an unrelated medication class and prevalent chronic use.

Multivariate logistic regressions were conducted to consider potentially interfering patient and tumor factors in our evaluation of relationships between anti-inflammatory medication use and cachexia incidence at diagnosis. Cachexia incidence at diagnosis functioned as the binary dependent variable. Patient factors included as covariates were age at diagnosis, sex, race, alcohol history, tobacco history, and Charlson Comorbidity Index. Tumor factors included were primary site and stage. Prior chronic medication use was included as a binary covariate, with null use denoting patients without prior use of any anti-inflammatory medications. Investigated. Overall survival differences between each medication use group versus the medication null group were estimated using the Kaplan-Meier method and evaluated statistically using log-rank testing for the total, cachectic, and noncachectic cohorts.

Associations between prior medication use and significant post-diagnosis weight loss in the non-cachectic cohort were evaluated through chi square testing and multivariate analyses. Multivariate analyses included all covariates from the previous multivariate analyses (age at diagnosis, sex, race, alcohol history, tobacco history, and Charlson Comorbidity Index), with the addition of systemic, surgical, radiation interventions as binary covariates. To visually represent weight loss progression in patients non-cachectic at diagnosis, Kaplan-Meier curves were created with significant post-diagnosis weight loss within as the event investigated and days from diagnosis to this event as the time variable.

All statistics were conducted with the alpha level of 0.05 defining statistical significance on SPSS Statistics for Mac Version 28.0.1.1 (International Business Machines, Armonk, NY).

## Results

### Patient Cohort and Cachexia at Diagnosis

After applying exclusion criteria, the database was composed of 3,180 consecutive cancer patients seen at the UT Southwestern Medical Center diagnosed between 1/1/2006 and 12/31/2013. Patient and tumor characteristics for the total cohort, as well as sub-cohorts defined by cachexia status at diagnosis, are presented in **Table 1**.

**Table 1:** Study population characteristics in total, cachectic, and non-cachectic cohorts.

Characteristic	Total cohort	No cachexia	Cachexia	Percent with cachexia
**N**	3180	2097	1083	34.06%
**Median age at diagnosis (IQR)**	62 (55-71)	63 (55-71)	62 (54-71)	
**Female (%)**	1355 (42.61%)	929 (44.30%)	426 (39.33%)	31.44%^‡^
**Race (%)**				
Asian or Pacific Islander	127 (3.99%)	82 (3.91%)	45 (4.16%)	35.43%
Black	758 (23.84%)	426 (20.31%)	332 (30.66%)	43.80% ^†^
Non-Hispanic White	1854 (58.30%)	1332 (63.52%)	522 (48.20%)	28.16%^‡^
Hispanic	363 (11.42%)	206 (9.82%)	157 (14.50%)	43.25%^†^
Other/unknown	78 (2.45%)	51 (2.43%)	27 (2.49%)	34.62%
**Primary tumor site (%)**				
Anal	92 (2.89%)	68 (3.24%)	24 (2.22%)	26.09%
Colorectal	623 (19.59%)	451 (21.51%)	172 (15.88%)	27.61%^‡^
Gastroesophageal	329 (10.35%)	143 (6.82%)	186 (17.17%)	56.53%^†^
Hepatobilliary	342 (10.75%)	259 (12.35%)	83 (7.66%)	24.27%^‡^
Pancreatic	267 (8.40%)	125 (5.96%)	142 (13.11%)	53.18%^†^
NSCLC	1369 (43.05%)	953 (45.45%)	416 (38.41%)	30.39%^‡^
Small cell lung cancer	158 (4.97%)	98 (4.67%)	60 (5.54%)	37.97%
**Tobacco (%)**	2267 (71.29%)	1477 (70.43%)	790 (72.95%)	34.85%
**Alcohol (%)**	1502 (47.23%)	1005 (47.93%)	497 (45.89%)	33.09%
**Charlson comorbidity index (%)**				
0	1034 (32.52%)	642 (30.62%)	392 (36.20%)	37.91%^†^
1	1049 (32.99%)	679 (32.38%)	370 (34.16%)	35.27%
2	541 (17.01%)	366 (17.45%)	175 (16.16%)	32.35%
3+	556 (17.48%)	410 (19.55%)	146 (13.48%)	26.26%^‡^
**Tumor grade (%)**				
1	142 (7.28%)	111 (8.42%)	31 (4.91%)	21.83%^‡^
2	1034 (53.03%)	731 (55.42%)	303 (48.02%)	29.30%^‡^
3	739 (37.90%)	459 (34.80%)	280 (44.37%)	37.89%^†^
4	35 (1.79%)	18 (1.36%)	17 (2.69%)	48.57%^†^
**Tumor stage (%)**				
1	541 (17.54%)	446 (21.86%)	95 (9.09%)	17.56%^‡^
2	531 (17.21%)	394 (19.31%)	137 (13.11%)	25.80%^‡^
3	879 (28.49%)	557 (27.30%)	322 (30.81%)	36.63%^†^
4	1134 (36.76%)	643 (31.52%)	491 (46.99%)	43.30%^†^

Significantly increased (†) or decreased (‡) cachexia incidence within row category relative to total cohort indicated (P<0.05). Significant (*) or non-significant (n.s.) differences of cachexia incidence between medication use groups indicated (P<0.05).

### Prior Medication Use and Cachexia Incidence at Diagnosis

**Figure 2:**
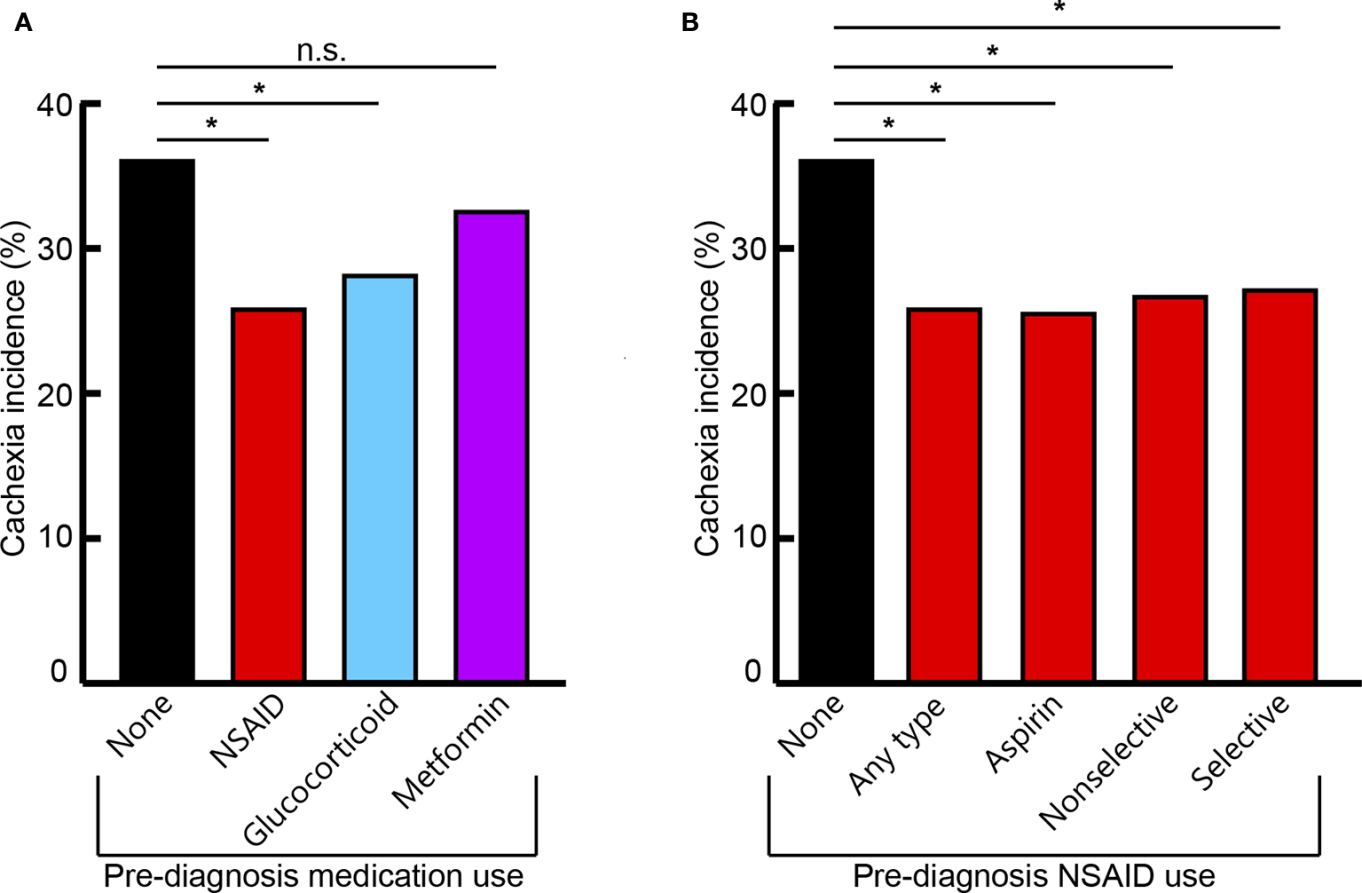
Cachexia incidence across cohorts of different prior medication use with overall anti-inflammatory medication class **(A)** and specific NSAID medication groups **(B)**. Nonselective NSAID medications include combined COX-1 and COX-2 inhibitors. Selective NSAID medications include specific COX-2 inhibitors. Significant (*) or non-significant (n.s.) differences of cachexia incidence between medication use groups indicated (P < 0.05).

The median time from the start of medication use to cancer diagnosis was 17 months. Patients with prior glucocorticoid use demonstrated an absolute decrease of 7.93% and a 21.94% relative decrease of cachexia incidence compared to patients who did not take anti-inflammatory medications prior to diagnosis (*P=0.0052*). Prior chronic use of any NSAID had 10.25% absolute and 28.36% relative decreases in cachexia incidence compared to patients who did not take anti-inflammatory medications prior to diagnosis (*P<0.0001*). Patients with prior chronic metformin use did not have significant reductions in cachexia incidence (*P=0.4685*; **Table 2**; **Figure 2A**).

**Table 2:** Categorical comparison of cachexia incidence in prior medication use cohorts and patients without prior anti-inflammatory medication use.

Prior medication use (n)	Patients with cachexia at diagnosis	P-value
**Total cohort (3180)**	1083 (34.06%)	
**No anti-inflammatory medication (2521)**	911 (36.14%)	
**Medication groups**		
Any glucocorticoid (319)	90 (28.21%)	***0.0053***
Any NSAID (479)	124 (25.89%)	***<0.0001***
Low-dose aspirin (301)	77 (25.58%)	***0.0003***
Other nonselective NSAIDs (157)	42 (26.75%)	***0.0172***
Selective COX-2 inhibitors (125)	34 (27.20%)	***0.0418***
Metformin only (95)	31 (32.63%)	***0.4685***

P values bolded if <0.05.

Specific NSAID medications included: patients with chronic use of low-dose aspirin; other nonselective COX-1 and COX-2 inhibitors such as indomethacin, diclofenac, and meloxicam; and selective COX-2 inhibitors such as celecoxib. Independently, all NSAID classifications demonstrated significantly decreased cachexia incidence at cancer diagnosis, with 8.94-10.56% absolute and 24.74-29.22% relative reductions, compared to patients without prior anti-inflammatory medication use (aspirin: *P=0.0003*; other nonselective: *P=0.0172*; selective: *P=0.0418*; **Table 2**; **Figure 2B**).

Multivariate analyses considering patient and tumor factors as covariates did not determine statistically significant changes in likelihood of cachexia incidence associated with glucocorticoid medication use (Odds ratio=1.046; 95% CI=0.778, 1.406; *P=0.7665*). Use of any NSAID prior to diagnosis was associated with decreased likelihood of cachexia incidence by 23.0% (Odds ratio=0.770; 95% CI=0.594, 0.998; *P=0.0481*; **Table 3**).

**Table 3:** Multivariate logistic regressions evaluating covariate associations with cachexia incidence at diagnosis including glucocorticoid and NSAID medication use groups.

Variable	Odds ratio (95% CI)	P-value
**Age at diagnosis**	1.012 (1.004, 1.020)	***0.0026***
**Female sex**	0.810 (0.678, 0.968)	*0.0206*
**Race**		
Asian or Pacific Islander	–	***<0.0001***
Black	1.602 (1.034, 2.482)	***0.0350***
Non-Hispanic White	0.719 (0.471, 1.098)	*0.1262*
Hispanic	1.508 (0.947, 2.401)	*0.0838*
Other/unknown	1.042 (0.528, 2.06)	*0.9049*
**Alcohol history**	0.924 (0.777, 1.099)	*0.3719*
**Tobacco history**	1.363 (1.100, 1.688)	***0.0046***
**Charlson Comorbidity Index**		
0	–	*0.0400*
1	0.979 (0.797, 1.201)	*0.8362*
2	0.848 (0.655, 1.098)	*0.2117*
3+	0.690 (0.524, 0.909)	***0.0084***
**Primary Tumor Site**		
Anal	–	***<0.0001***
Colorectal	0.762 (0.435, 1.335)	*0.3420*
Gastroesophageal	2.491 (1.400, 4.430)	***0.0019***
Hepatobiliary	0.735 (0.406, 1.332)	*0.3108*
Pancreatic	2.814 (1.547, 5.118)	***0.0007***
NSCLC	0.725 (0.420, 1.252)	*0.2483*
Small-cell lung cancer	0.824 (0.431, 1.575)	*0.5590*
**Tumor stage**		
I	–	***<0.0001***
II	1.232 (0.882, 1.719)	*0.2208*
III	2.309 (1.728, 3.086)	***<0.0001***
IV	3.290 (2.477, 4.369)	***<0.0001***
**Prior glucocorticoid use**	1.046 (0.778, 1.406)	*0.7665*
**Prior NSAID use**	0.770 (0.594, 0.998)	***0.0481***

P values bolded if <0.05.

### Survival

Prior to the review of the effect of anti-inflammatory medications on non-cachectic- and cachectic-specific survival, median survival time was determined to be 23 months (95% CI=21.161, 24.839) in the total patient cohort, 14 months (95% CI=12.659, 15.341) in patients with cachexia at diagnosis, and 31 months (95% CI=26.797, 35.203) for patients without cachexia at diagnosis. Patients with prior use of glucocorticoid medications had median survival times significantly lower than their medication-null counterparts in the non-cachexia group (20 vs 34 months; *P=0.0018*) and did not reach a significant difference within the cachexia cohort (16 vs 13 months; *P=0.89*61). Patients with prior NSAID use had median survival times significantly lower than their medication-null counterparts in the non-cachexia group (26 vs 34 months; *P=0.0149*) and did not reach a significant difference for the cohort of patients with cachexia at diagnosis (13 vs 13 months; *P=0.1554*). Similar findings were observed across specific NSAID groups and are further demonstrated in **Table 4**.

**Table 4:** Log-rank analysis comparing overall survival in medication groups to no anti-inflammatory medication groups across all, cachectic, and non-cachectic cohorts.

	All patients (n=3180)		Cachectic patients (n=1083)		Non-cachectic patients (n=2097)	
Prior medication use	Median time (95% CI)(months)	P-value log-rank	Median time (95% CI)(months)	P-value log-rank	Median time (95% CI)(months)	P-value log-rank
**All patients**	23 (21.161, 24.839)		14 (12.659, 15.341)		31 (26.797, 35.203)	
**No anti-inflammatory medication**	23 (20.780, 25.220)		13 (11.564, 14.436)		34 (28.401, 39.599)	
**Medication groups**						
Any glucocorticoid	19 (14.030, 23.970)	***0.0252***	16 (8.588, 23.412)	*0.8961*	20 (13.708, 26.292)	***0.0018***
Any NSAID	21 (16.922, 25.078)	***0.0449***	13 (8.646, 17.354)	*0.1554*	26 (18.86, 33.14)	***0.0149***
Low-dose aspirin	21 (16.048, 25.952)	*0.0610*	10 (6.946, 13.054)	***0.0285***	25 (15.084, 34.916)	*0.0781*
Other nonselective NSAIDs	19 (11.379, 26.621)	*0.0606*	13 (6.991, 19.009)	*0.3157*	26 (14.818, 37.182)	***0.0337***
Selective COX-2 inhibitors	22 (14.415, 29.585)	*0.5529*	10 (2.857, 17.143)	*0.6695*	26 (15.873, 36.127)	*0.3469*

P values bolded if <0.05.

### Prior Anti-Inflammatory Use and Incidence of Significant Post-Diagnosis Weight Loss

Of the 2003 patients that did not meet criteria for cachexia at cancer diagnosis, 44.43% met the cutoff of 5% weight decrease within 6 months post-diagnosis. When evaluated based on prior medication use, 42.06% of non-cachectic patients who did not take any anti-inflammatory medication prior to diagnosis met criteria for weight loss within 6 months post-diagnosis. This was significantly lower than the 52.11% (*P=0.0006*) and 51.75% (*P=0.0059*) of non-cachectic patients with prior NSAID or glucocorticoid use, respectively, that met the post-diagnosis weight loss cutoff. Similar findings were observed across specific NSAID groups (**Table 5**).

**Table 5:** Categorical comparison of significant post-diagnosis weight loss incidence (in patients without cachexia at diagnosis) between prior medication use groups and anti-inflammatory medication null patients.

Prior medication use category (N)	Patients that reached significant weight loss within 6 months of cancer diagnosis (%)	P-value
**No anti-inflammatory medication (1517)**	638 (42.06%)	
**Medication groups**		
Any glucocorticoid (228)	118 (51.75%)	***0.0059***
Any NSAID (355)	185 (52.11%)	***0.0006***
Low-dose aspirin (224)	113 (50.45%)	***0.0180***
Other nonselective NSAIDs (115)	58 (50.43%)	*0.0799*
Selective COX-2 inhibitors (91)	53 (58.24%)	***0.0025***

P values bolded if <0.05.

Within the same cohort of 2003 patients who did not meet cachexia criteria at diagnosis, multivariate analyses considering patient, tumor, and treatment factors, prior use of any glucocorticoid medication associated with a 45.2% (Odds ratio=1.452; 95% CI=1.065, 1.979; *P=0.0183*) increased likelihood of meeting criterion for significant post-diagnosis weight loss by 6 months. Prior use of any NSAID medication similarly predicted for 41.1% (Odds ratio=1.411; 95% CI=1.082, 1.840; *P=0.0110*) increased likelihood of reaching the same criterion (**Supplementary Table 2**).

**Figure 3:**
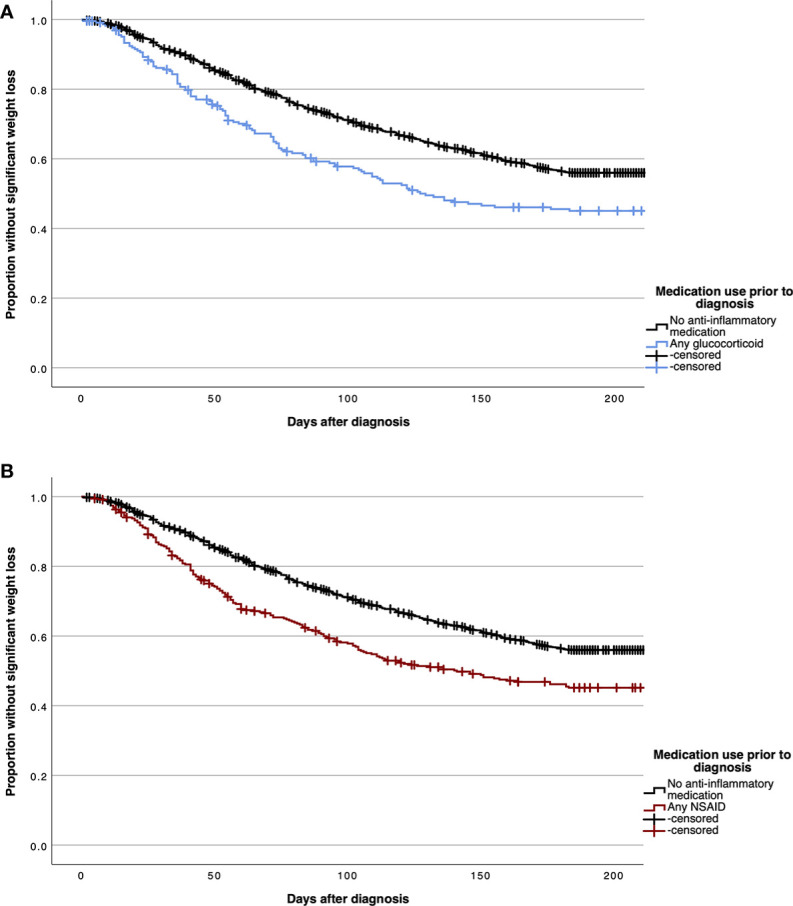
Kaplan Meier curves demonstrating time to significant post-diagnosis weight loss in patients without cachexia at diagnosis for patients with prior chronic use of any glucocorticoid **(A)** and any non-steroidal anti-inflammatory drug (NSAID) **(B)** medications.

**Figures 3A, B** demonstrate Kaplan-Meier curves for time-to-cachexia for patients with prior chronic use of any glucocorticoid or NSAID medication compared to patients without prior anti-inflammatory medication use.

## Discussion

### Overall Findings

In this study, we investigated relationships between prior chronic use of NSAID and glucocorticoid medications with cachexia at cancer diagnosis. Multivariate analyses demonstrated a particularly relevant 25% reduction in likelihood of cachexia presentation associated with the NSAID medication class that was independent of potentially confounding patient and tumor characteristics, including comorbidities. Furthermore, our survival and post-diagnosis cachexia analyses are consistent with a delay in cachexia progression attributable to these medications. Although retrospective studies cannot establish causality and are vulnerable to sampling bias, we applied statistical rigor to our dataset through the application of multiple analytical methods and subdivided our cohort to decrease bias and reduce error in our investigation. The novel focus of this study on use of NSAIDs and glucocorticoids prior to cancer treatment intervention primarily highlights NSAIDs for further investigation of therapeutic potential early in cachexia-specific cancer management.

### Bridging Cachexia Pathophysiology and Therapeutic Strategy

Our decision to further investigate the therapeutic potential of NSAID and glucocorticoid medications stemmed from the mechanistic link between these medications and cachexia, as well as further evidence from animal models (17) and clinical trials supporting the use of these medications to attenuate weight loss in the setting of advanced malignancy (**Supplementary Table 1**) (18–22). In our comprehensive literature review, we were unable to identify any prior attempts at evaluating potential benefits of these medications in early or even sub-clinical stages of cachexia progression.

The central mechanism of NSAIDs is inhibition of COX-1 and COX-2 enzymes, which are expressed in almost every human tissue (23). Although COX-1 function is largely stable as a mediator of various housekeeping functions, COX-2 function can vary widely due to upregulation in states of inflammation. COX-2 upregulation has been demonstrated to contribute to tumor growth and metastatic spread by promoting angiogenesis and immune evasion (24–28). Many of the pathologic manifestations of cancer cachexia are understood to arise from inflammatory signaling dependent on upregulated COX production of prostaglandins.

Pivotal to progression of cachexia is the combination of hypophagia and excessive energy expenditure that promote a debilitating state of catabolism. Although multifactorial in nature, hypophagia observed in cancer cachexia is understood to be primarily generated through the dysregulation of hypothalamic-pituitary axis function by characteristic inflammatory cytokines including TNF-α, IL-1β, and IL-6 (29, 30). Prostaglandins propagate the generation of these cytokines in the periphery and modulate the integration of signaling centrally within the hypothalamus and brainstem (31–34). Additionally, this inflammatory signaling disturbs thermoregulatory control, contributing to maladaptive energy expenditure. Prostaglandins have also been implicated in the metabolically inefficient sympathetic overactivation observed in cachexia (35–37). At sites of adipose stores, prostaglandins mediate the induction and recruitment of thermogenic brown adipose tissue, further contributing to energy wasting in cachexia in a mechanism attenuated by COX-2 inhibition in murine models (38).

In addition to negative overall metabolic flux, functional decline in cachexia is largely attributable to skeletal muscle wasting from systemic catabolic and inflammatory signaling (39). In multiple murine tumor models, COX inhibition has demonstrated efficacy at preventing cachectic skeletal muscle degradation, furthering the relevance of NSAID function in this syndrome (40, 41).

### Clinical Relevance

Cancer cachexia remains a critically undertreated component of disease burden across almost every cancer type. Factors contributing to this deficiency include limited options available for management and identification. Although cancer patients are well known to undergo weight loss from treatment-induced anorexia, cachexia uniquely intensifies energy expenditure through malignant metabolic and inflammatory mechanisms.

The evaluation of relationships between retrospective medication was carefully considered, as chronic use of any anti-inflammatory medication implies a comorbid condition. This implication prompted our application of multivariate analyses to simultaneously control for comorbidity, as represented by Charlson Comorbidity Index in our cohort, in addition to a multitude of patient and tumor factors. The significant 23% decreased likelihood of cachexia incidence attributable to prior NSAID use interpreted through this method suggests these medications might currently be underutilized in cachexia management and prevention.

On initial evaluation, our results demonstrated a survival detriment observed across medication use groups primarily in patients without cachexia at diagnosis. Further evaluation of non-cachectic patients revealed an approximate increase of 10% in the incidence of significant weight loss within 6 months post-diagnosis in patients with prior use of NSAIDs or glucocorticoids. In this cohort of patients without cachexia at diagnosis, multivariate analyses considering interfering patient, tumor, and treatment factors found significant increases in likelihood of post-diagnosis weight loss of more than 40% in NSAID and glucocorticoid use groups. If these medications indeed suppressed the inflammatory ramifications of cachexia-inducing tumor phenotypes prior to diagnosis, it is possible that the reduction of non-cachectic survival represents a progression of cancers which would have classified patients as having cachexia at diagnosis in the absence of prior NSAID or glucocorticoid use. Ultimately, the observed reduction in overall survival within non-cachectic patients with prior NSAID or glucocorticoid use in combination with their increased burden of post-diagnosis weight change potentially represents a delay in cachexia progression by these medications.

### Conclusion

The findings of this study substantiate further investigation into cachexia-specific intervention with these medications, primarily NSAIDs, early in cancer development. The clinical outcomes we observed are consistent with the biomolecular reasoning that cachexia progression would possess vulnerability to this medication type, which specifically mitigates inflammatory mechanisms of the syndrome not attributable to the body tissue wasting observed consequently to oncologic therapies. Individuals with elevated cancer risk or unexplained weight loss may benefit from these medications prophylactically, as they could potentially blunt cachexia development before patients are burdened by sequelae from cancer progression and treatment. Deciphering the clinical merit contributed by medications with anti-inflammatory properties in this context can alter their prioritization in treatment protocols and physician judgement for managing patients with chronic conditions simultaneously at risk for developing cachexia-inducing tumors.

## Data Availability Statement

The patient data supporting the conclusions of this article is deidentified through statistical interpretation. The raw data can be made available, without undue reservation, upon reasonable request to the corresponding authors.

## Author Contributions

SO, PI, and RI designed and conceptualized study. SO, BG, and AG participated in data curation. SO conducted formal analyses. PI RI contributed to funding, project administration, and supervision. SO wrote the original draft. All authors participated in review and editing of this project for final publication. All authors contributed to the article and approved the submitted version.

## Funding

This work was supported by National Institutes of Health [P30 CA142543]; Burroughs Wellcome Fund Career Awards for Medical Scientists [1019692]; American Cancer Society grant [133889-RSG-19-195-01-TBE]; Cancer Prevention and Research Institute of Texas [RP200170]; V Foundation Scholar Award [V2019-014]; and American Gastroenterological Association Scholar Award [2019AGARSA3].

## Conflict of Interest

The authors declare that the research was conducted in the absence of any commercial or financial relationships that could be construed as a potential conflict of interest.

## Publisher’s Note

All claims expressed in this article are solely those of the authors and do not necessarily represent those of their affiliated organizations, or those of the publisher, the editors and the reviewers. Any product that may be evaluated in this article, or claim that may be made by its manufacturer, is not guaranteed or endorsed by the publisher.
