# Supplementary material for: Associations of Prior Chronic Use of Non-Steroidal Anti-Inflammatory Drugs (NSAIDs) and Glucocorticoids With Cachexia Incidence and Survival
Source: Front Oncol. 2022 Jun 7;12:922418. doi: 10.3389/fonc.2022.922418 (PMC9210667; doi:10.3389/fonc.2022.922418)
Supplement: Supplementary file 1 [file DataSheet_1.pdf]

Supplementary Table 1: Prior studies involving anti-inflammatory medications to manage cachexia in advanced malignancies.

Authors, year, study design	Patient population of cohort (duration of study)	Treatment arms (n)	Statistically significant notable results
Maccio et al. (2012), clinical trial	Patients with advanced-stage gynecological (ovarian, endometrial, cervical) cancer. (4 months)	-Megestrol acetate 320 mg/day + celecoxib 300 mg/day + carbocysteine 2.7 g/day + carnitine 4g/day + lipoic acid 600 mg/day (61)<br><br>Megestrol acetate 320 mg/day (control) (63)	Combinational therapy associated with improved lean body mass, leptin levels, resting energy expenditure, fatigue, and quality of life scores, with decreased serum levels of cachexia associated markers including IL-6, TNF-a, C-reactive protein, and reactive oxygen species.
Mantovani et al. (2010), clinical trial	Advanced cancer patients with ≥ 5% pre-illness weight loss in 3-6 months prior to cancer diagnosis with abnormal values of inflammatory cytokines. (4 months)	-Celecoxib 300 mg/day (24)	Improved lean body mass, grip strength, quality of life, ECOG performance status[1], Glasgow prognostic score[2] at end of treatment with decreased levels of TNF-a measured.
Lundholm et al. (2004), retrospective case control	Patients with generalized malignant cancer without interventional treatment in 4-6 months prior to evaluation. (24 months)	Indomethacin treatment (151)<br><br>No indomethacin or other non-steroidal anti-inflammatory (NSAID) treatment (control) (145)	Resting energy expenditure, total body fat loss, C-reactive protein and erythrocyte sedimentation rate values decreased in indomethacin treatment group relative to control, along with body weight increase.
McMillan et al. (1997), clinical trial	Locally advanced or metastatic gastrointestinal cancer patients with ≥ 5% weight loss (without reported dysphagia or obstruction) receiving only supportive care. (16 weeks)	Ibuprofen 1200 mg/day + megestrol acetate 480 mg/day (22)<br><br>Megestrol acetate 480 mg/day + placebo (control) (19)	Improvement of body weight in ibuprofen-treated group, compared to megestrol acetate alone, at both measurement intervals.
Lundholm et al. (1994), clinical trial	Terminally ill cancer patients more than 6 months after their last cancer treatment with insidious or ongoing weight loss attributable to disease. (until death)	Indomethacin 100 mg/day (45)<br><br>Prednisolone 20 mg/day (45)<br><br>Placebo (control) (45)	Indomethacin treatment associated more than double mean survival time as placebo group. Indomethacin or prednisolone treatment associated with improved Karnofsky Index[3]. Grip strength improved in prednisolone group.

[1] ECOG performance status assesses how disease affects patients' activities of daily living determined by Eastern Cooperative Oncology Group
[2] Glasgow prognostic score utilizes C-reactive protein and albumin levels to assess inflammation levels to predict cancer outcomes
[3] Karnofsky Index classifies patients according to their functional impairment

Supplementary Table 2: Multivariate logistic regressions evaluating covariates to incidence of 5% weight loss within 6 months post-diagnosis for medication use groups evaluated with medication null group.

	Any glucocorticoid		Any NSAID	
**Variable**	**Odds ratio (95% CI)**	**P-value**	**Odds ratio (95% CI)**	**P-value**
**Age at diagnosis**	1.009 (1.000, 1.019)	*0.0615*	1.011 (1.001, 1.02)	***0.0277***
**Female sex**	0.977 (0.785, 1.216)	*0.8353*	0.981 (0.794, 1.213)	*0.8600*
**Race**				
Asian or Pacific Islander	-	***0.0009***	-	***0.0112***
Black	0.549 (0.313, 0.963)	***0.0365***	0.530 (0.309, 0.908)	***0.0208***
Non-Hispanic White	0.964 (0.569, 1.633)	*0.8926*	0.828 (0.5, 1.37)	*0.4617*
Hispanic	0.654 (0.358, 1.193)	*0.1665*	0.653 (0.367, 1.164)	*0.1482*
Other/unknown	0.902 (0.383, 2.121)	*0.8125*	0.896 (0.399, 2.009)	*0.7890*
**Alcohol history**	0.92 (0.743, 1.139)	*0.4447*	0.948 (0.772, 1.165)	*0.6138*
**Tobacco history**	0.925 (0.714, 1.200)	*0.5585*	0.917 (0.714, 1.178)	*0.4984*
**Charlson Comorbidity Index**				
0	-	***0.0105***	-	***0.0211***
1	1.333 (1.023, 1.736)	***0.0334***	1.396 (1.081, 1.803)	***0.0106***
2	1.557 (1.124, 2.159)	***0.0078***	1.402 (1.028, 1.913)	***0.0327***
3+	1.662 (1.184, 2.334)	***0.0033***	1.555 (1.122, 2.156)	***0.0080***
**Primary tumor site**				
Anal	-	***0.0400***	-	***0.0036***
Colorectal	0.722 (0.377, 1.382)	*0.3254*	0.66 (0.354, 1.234)	*0.1932*
Gastroesophageal	1.166 (0.574, 2.369)	*0.6702*	0.972 (0.491, 1.925)	*0.9346*
Hepatobiliary	0.767 (0.392, 1.500)	*0.4383*	0.606 (0.315, 1.166)	*0.1337*
Pancreatic	1.083 (0.511, 2.296)	*0.8353*	1.097 (0.532, 2.265)	*0.8015*
NSCLC	0.715 (0.383, 1.336)	*0.2935*	0.591 (0.324, 1.078)	*0.0864*
Small cell lung cancer	0.411 (0.183, 0.923)	***0.0313***	0.325 (0.148, 0.711)	***0.0049***
**Tumor stage**				
I	-	***0.0231***	-	***0.0279***
II	1.304 (0.915, 1.858)	*0.1424*	1.408 (1.000, 1.982)	*0.0500*
III	1.629 (1.152, 2.304)	***0.0058***	1.644 (1.172, 2.306)	***0.0040***
IV	1.659 (1.162, 2.369)	***0.0053***	1.573 (1.116, 2.216)	***0.0097***
**Systemic treatment**	1.324 (1.017, 1.722)	***0.0367***	1.307 (1.008, 1.694)	***0.0437***
**Surgical treatment**	0.965 (0.738, 1.262)	*0.7941*	1.02 (0.787, 1.321)	*0.8835*
**Radiation treatment**	0.837 (0.647, 1.084)	*0.1775*	0.789 (0.614, 1.013)	*0.0635*
**Medication use**	1.452 (1.065, 1.979)	***0.0183***	1.411 (1.082, 1.840)	***0.0110***
